# Human difference in the genomic era: Facilitating a socially responsible dialogue

**DOI:** 10.1186/1755-8794-3-20

**Published:** 2010-05-26

**Authors:** Sarah Knerr, Edward Ramos, Juleigh Nowinski, Keianna Dixon, Vence L Bonham

**Affiliations:** 1 National Human Genome Research Institute, National Institutes of Health, 31 Center Drive, Bethesda, MD 20892 USA; 2 Center for Research on Genomics and Global Health, National Institutes of Health, 12 South Drive, Bethesda, MD 20892 USA; 3 Institute for Public Health Genetics, University of Washington, Box 357236, Seattle, WA 98195 USA; 4 Office of the Assistant Secretary for Health, Department of Health and Human Services, 200 Independence Avenue, S.W., Washington, D.C. 20201 USA; 5 Columbia University, 2960 Broadway, New York, NY 10027 USA

## Abstract

**Background:**

The study of human genetic variation has been advanced by research such as genome-wide association studies, which aim to identify variants associated with common, complex diseases and traits. Significant strides have already been made in gleaning information on susceptibility, treatment, and prevention of a number of disorders. However, as genetic researchers continue to uncover underlying differences between individuals, there is growing concern that observed population-level differences will be inappropriately generalized as inherent to particular racial or ethnic groups and potentially perpetuate negative stereotypes.

**Discussion:**

We caution that imprecision of language when conveying research conclusions, compounded by the potential distortion of findings by the media, can lead to the stigmatization of racial and ethnic groups.

**Summary:**

It is essential that the scientific community and with those reporting and disseminating research findings continue to foster a socially responsible dialogue about genetic variation and human difference.

## Background

The accomplishments of the Human Genome Project have ushered in a new genomic era. The mission to decipher our genetic blueprint is being succeeded by a mission to understand the interplay between genetic factors, the environment, and human traits in order to promote health through novel treatments and preventative strategies. Genetic differences that exist between individuals and groups are a key piece to this complex puzzle. Referred to as human genetic variation (HGV), these differences can be single base changes (single nucleotide polymorphisms, or SNPs) as well as large stretches of DNA that are deleted, duplicated, or inverted (i.e., structural or copy number variations) [1-4]. Knowledge gained from continued research into HGV holds tremendous promise for the personalization of medicine by linking individual genetic information--in addition to non-genetic risk factors--to disease prevention and drug response [5]. The expected benefit of individualizing medical treatments in this way is a more efficient prediction, prevention, diagnosis, and treatment of human disease.

The recent and rapid technological advances in how we gather genomic information are driving forces of scientific progress in the genomic era. The development of "next generation sequencing" techniques, for example, has caused the price of full genome sequencing to fall rapidly. The pace at which the field is moving makes predicting future costs difficult, as estimations are quickly outdated [6]. For example, the sequencing of Craig Venter's genome in 2007 [7] and James Watson's genome [8] in 2008 took approximately two months and cost just under $1 million dollars; however, Dr. James Lupski of Baylor College of Medicine recently reported sequencing his entire genome for $50,000 [9]. This figure will likely continue to decrease in the near future as indicated by Complete Genomics' efforts towards a $5000 genome [10] and the continued interest in personal genomics [11]. Removing cost barriers to obtaining whole-genome data has dramatically expanded the range of research questions that geneticists can explore. The increased speed and accuracy of genetic analysis has made genome-wide association studies (GWAS)--statistical associations between known genetic variants and quantitative traits--a powerful research technique. As a result, the number of genomic variants associated with diseases and other human characteristics has been climbing, with the pace of discovery quickening dramatically in the past year [12,13]. As of July 27^th ^2009, 369 papers describing 1682 SNPs associated with diseases ranging from type I diabetes [14] to narcolepsy [15] and traits such as hip bone size [16] and height [17] have been published in peer-reviewed journals http://www.genome.gov/gwastudies. Most variants identified through GWAS explain only a small percentage of the associated trait's heritability and must also be considered within the context of environment. Translating this type of genetic information into preventative health and medical interventions, especially with little existing evidence-based research relevant to each disease, remains an elusive goal.

Despite uncertainty about the ultimate clinical utility of many new genetic discoveries, there is no doubt that we are in an era of large-scale genomic studies which aim to understand the intricate details of human traits across different groups and between individuals. Significant progress has been made in understanding genetic differences by comparing individuals on the basis of genotype data rather than social classifications [18]. However, uncovering these genetic differences has raised important questions about how to articulate the complex patterns of human genetic variation that scientists are beginning to uncover. Allele frequency can be correlated with geographic ancestry, meaning certain variants may be more common among individuals whose predecessors lived in the same area, but translating this type of information into identifiable groups that are currently meaningful in a clinical setting has been difficult. Frequently, geneticists describe "populations" for which their results apply, but this term can be ambiguous and imprecise and often refers to current social categories of race and ethnicity, which are not genetically distinct. Moreover, throughout history, science has been misused to demonstrate differences between human groups and rationalize discrimination. Therefore, it is essential for current and future research on genetic variation to consider the lessons learned from past studies of human difference and recognize the current social and political environment in which results will be contextualized. The caution of accuracy and precision in reporting and interpreting genetics research spans the scientific, media, and public communities. However, the suggestions laid forth are geared specifically to genetics and genomics researchers as the study of human genetic variation continues to advance.

## Discussion

### Communicating human genetic variation

Two specific examples can help illustrate how research conceived as scientifically sound and value-independent can lead to the misrepresentation of the role of genetic factors in human traits. The first example is a hotly debated hypothesis that emerged from research on the role of salt retention in heart disease in African Americans beginning in the 1980s. Termed the "Slavery Hypothesis", the original hypothesis is attributed to Clarence Grim, who published an article titled, *Biohistory of slavery and blood pressure in blacks today. A hypothesis*, with Thomas Wilson in 1991 [19]. The hypothesis asserts that African Americans--as the descendents of predominately West Africans who survived the transatlantic slave trade--possess genetic variants that predispose them to hypertension and heart disease in the present-day environment at a higher rate than the rest of the United States population. Briefly, the hypothesized mechanism supporting this argument is that the conditions of the middle passage created a genetic bottleneck, selecting for genes protective against "...sodium depletion from low sodium intake or high sodium loss attributable to sweating, diarrhea and vomiting, especially when combined with conditions of high environmental temperature and high physical work demands" [20].

Critics have attacked many aspects of the hypothesis, specifically its description of the physiology and pathology of hypertension to its interpretation of evolutionary biology theory [21-23]. Despite the questionable scientific and historical support, the Slavery Hypothesis--and its various reincarnations--are frequently cited in papers about cardiovascular disease and receive ample positive attention in the public press. For example, the Slavery Hypothesis was promoted as scientific fact during an episode of The Oprah Winfrey Show in May 2007 and by Harvard economist Roland Fryer during his appearance on CNN's documentary *Black in America *in August 2008. In the words of Thomas A. LaVeist, Ph.D., Director of the Center for Health Disparities at the Bloomberg School of Public Health at Johns Hopkins University, "This bogus theory just won't seem to die. Even though public health researchers have discredited the theory it continues to be promoted by people who are not knowledgeable about the field" [24].

Research completed on brain size provides a second illustrative example. In 2005, Bruce Lahn and colleagues published two papers suggesting that mutations in the brain developmental genes *abnormal spindle-like microcephaly associated *(*ASPM*) and *microcephalin*, were under positive selection (i.e., provide an advantage to individuals who possess them). They reported that such evidence of selection signaled that the brain is still evolving and that the two adaptive variants occur at a higher rate in Eurasian than in African populations [25,26]. Additionally, they speculated that the emergence of these variants was correlated with the spread of domestication, the development of cities, and the emergence of written language [25,26]. The popular press depicted this research as supporting a link between brain size, intelligence, genetics, and race, despite criticism of the study's findings and their interpretation from other genetic researchers and follow-up studies with contradictory results [27,28]. Heated discussion of these studies, their results, and how they were communicated to and interpreted by the public have continued [29] even though Lahn published subsequent work that concluded early genetic findings do not support a direct link to cognition [30].

The previous examples share two common themes. First, both describe scientists who strongly believed they were reporting unbiased scientific fact and did not believe their research was perpetuating racism. Grim and Robinson stated that they "...do not feel that accusations of racism belong in a scientific discussion of populations that differ in physiology or gene frequency" [20]. Lahn has criticized scientists that, in his view, "start with a political agenda and fit the evidence to that", going on to say that any type of bias, "takes credibility from the antiracist program I agree with...If someday we discover there are genetic differences in cognitive abilities, would that mean that racism is justified?" [31]. The second common theme from these examples is their ability to lend support to the ideology that essential, meaningful differences in fitness and ability exist between human groups. These cases illustrate how, despite best intentions, studies that link genetic variation and human difference can lead to unsubstantiated and often harmful assertions in peer-reviewed literature and mainstream media.

Scientific efforts to explore differences between groups can easily become value-laden and misused as "evidence" to support a certain social perspective. Past and present stereotypes and existing social structures can both overtly and unconsciously lead to the portrayal of minority groups as fundamentally different-- interpreted often as abnormal, defective, or overly susceptible --when compared to majority groups. In the case of the Slavery Hypothesis, epidemiologists Jay S. Kaufman and Susan A. Hall have documented the prevalence of article titles such as "*Hypertension in Blacks. Is it a Different Disease?*" and statements such as,"...hypertension in black subjects is linked to abnormalities of control of intracellular sodium," in published articles. Referencing Richard Cooper and Charles Rotimi [32], Kaufman and Hall state that other ethnic groups with hypertension prevalence comparable with that of African-Americans, notably the Finns, are not described as harboring genetic defects or physiologic abnormalities [33]. Unfortunately, the misrepresentation of scientific findings through misrepresented language persists. In 2008, a study reported that a variant in the Duffy antigen receptor for chemokines conferring resistance to malaria was associated with increased odds of having HIV-1 for the African Americans in the study's sample population [34]. Subsequent press headlines that reported this finding read: "Genetic Trait Makes Africans Especially Prone to HIV Infection" [35], "Anti-Malaria Mutation in Blacks Promotes HIV Infection" [36], and "Anti-malaria gene 'makes Africans more susceptible to HIV'" [37]. Presenting genetic findings in such a way--with even subtle negative connotations--can have wide-ranging effects. For example, after Lahn's work on brain size was publicized, National Review Online columnist John Derbyshire wrote that it implied, "our cherished national dream of a well-mixed and harmonious meritocracy...may be unattainable" [38]. Though this statement is an extreme case of research findings being applied broadly out of context, it illustrates how discoveries of genetic variation between human groups can have wide-ranging social implications.

### Accurate reporting of research findings

The potential for misinformation about the complex relationships between genetic factors, environmental factors, and human phenotypes increases as the number of studies exploring human genetic variation and complex traits, such as human behavior, continues to climb. For example, recent studies have explored genetic variation as it relates to delinquent peer affiliation in males [39], chaotic family environments [40], and transsexualism [41]. The consequences of comparing across different groups with respect to these charged issues are largely unknown, as most studies have been carried out in a single population. The general public's lack of understanding about even the most basic genetics concepts suggest that such studies have the potential to perpetuate misconceptions about HGV, likely stigmatizing groups. However, recent calls for revising the genetics curriculum to focus on complex traits might help improve public understanding [42].

At the core of many of the controversies outlined above is the poor representation or misinterpretation of research outcomes and conclusions. Nuanced statistics, probabilities, percentages, and the like are quickly boiled down to a "yes" or "no" when Population A, for example, is more susceptible to a disease than Population B. The implications of such simplifications are far greater given that the subjects associated with these conclusions are not yeast, or mice, but humans. Concern arises when Population A no longer reflects a genotype, but a social grouping, which quickly loses its utility as an accurate biological indicator. Statements along the lines of "Latinos are more susceptible," or "African Americans are less susceptible" to disease, drug response, or behavioral traits do not translate into "all" Latinos or "all" African Americans but, rather, a certain fraction of these populations. We must issue caution to researchers involved in this new wave of research on HGV that the impact of their findings are not only scientific, but have social, ethical and clinical implications (e.g., diagnostic implications) [43,44]. When researchers fail to consider or accurately describe the nuances in associations they observe, they give the impression that they fail to see, or choose to dismiss, the social ramifications of their claims.

Realizing this concern, the issue then becomes what can the scientific community do to facilitate a responsible, positive dialogue in the genomic era? A researcher might begin by considering the basis for examining his or her particular hypothesis. In exploring a genetic contribution to an apparent health difference, is the milieu within which this health difference is manifested being given proper attention? As with any research, confounding factors should be acknowledged and controlled for, if possible. Considering potential genetic variation in a silo rather than within the context of environment and other factors would be an inaccurate reflection of our current understanding of biological processes. As Yen-Revollo and colleagues describe with respect to individual drug response, commonly used racial labels are both insufficient and inaccurate representations of inferred genetic clusters in a heterogeneous population [45]. Thus, researchers should give thought to how the different populations in a study have been defined, specifically asking: have socially determined definitions of race and ethnicity been used and what are the implications for analyzing data and reporting results?

These concerns are not meant to halt the progress of human genetic variation research, but rather meant to convey a thoughtful "proceed with care" to ensure the best and most appropriate interpretation and ultimate utility of results for understanding human difference and improving human health. To be fair, the burden of responsibility does not fall squarely on researchers, as journal editors and the media play an essential role in how genetic findings are presented. Scientific journals are the first link in the dissemination of information--selectively culling research findings and presenting them to the larger scientific community. From scientific journals, research results then move into the larger public arena through science and public interest coverage in the mainstream media. Researchers should give thought to how the general public and mass media might interpret the research findings. Thinking through the questions mentioned above might preemptively limit the possibility that results will be misinterpreted or taken out of context.

Writers and researchers should maintain a two-way dialogue during the process of publishing web and print articles on genetic findings for the general public--both are responsible for clarifying misreporting of genetic findings and presenting corrections in a timely manner. Individual researchers have the responsibility to contact reporters who inaccurately present their work and offer corrections. The internet has a potential for a wider dissemination of information than any other current arena of communication, any stigmas and prejudices that are provoked by research findings related to human genetic variation can be perpetuated faster than ever through this avenue. Critics of science reporting have cited a proliferation of inaccurate coverage, but studies have shown that journalists do not necessarily inaccurately report findings as much as they fail to properly translate the language of science vocabulary to lay vocabulary [46,47]. The difficulty of translating scientific vocabulary to lay language and the quest to produce articles that cater to human interest contributes to the use of sensationalist language that can overemphasize the significance of genetic findings and increases the perception of unbalanced reporting. Scientists should continue to recognize the importance of accurate reporting so that journalists writing for the general public avoid inappropriately highlighting the role of genetic factors in differences between human populations as well as report on the potential limitations and social implications of the research.

## Summary

Advancements in genomic research have provided the scientific community with a deeper appreciation for the complexity of human genetic variation and its role in health and disease. The scenarios presented in this paper describe unintended consequences of attempts to describe genetic difference (perceived or real) between groups, which could potentially lead to the creation or emphasis of disparaging stereotypes and stigmas. However, identifying a problem often takes much less energy than implementing a solution. Here we suggest three ways that human genetic scientists may catalyze a transition towards increasing social responsibility in the field.

First, researchers should encourage critical reading of scientific papers and their corresponding media representations not only among colleagues, but also among their families, friends, and peers outside of human genetics. Most importantly, scientists should emphasize special consideration of how study populations are defined, the heterogeneity that may exist in these definitions, and the resulting implications for presentation and application of results. Encouraging informed and analytical consumption of research findings will facilitate public dialogue about the relationship between human genetic variation and social identity. Second, human genetic researchers must emphasize the responsibility of learning from past mistakes. This adage is not only applicable to the field human genetics, but the increasing profile of genetic and genomics research makes education about past misuses of genetic information particularly urgent. Finally, the current vanguard of scientists should commit to cultivating a generation of human genetic researchers who are actively engaged in discussions of the social implications of their work--for whom the responsible design, implementation, and dissemination of their research is a driving priority. This could be done by actively including instruction about relevant past misuses of science and how to prevent them as a part of scientific mentorship. The genome era has already provided tremendous insight into the etiology of a number of disorders and created new and exciting opportunities for researchers to directly investigate human disease at the population level. By following these and other guidelines [48,49], current and future scientists can promote a creed of personal responsibility in human genetics research that will allow the benefits of this era of discovery to be fully realized.

## List of Abbreviations

HGV: human genetic variation; SNP: single nucleotide polymorphism; GWAS: genome-wide association studies.

## Competing interests

The authors declare that they have no competing interests.

## Authors' contributions

SK and ER contributed equally to the writing of the first and final draft of this manuscript. JN, KD, and VLB contributed to the text and all authors contributed to the conception and design. All authors contributed editorial comments, read and approved the final manuscript.

## Pre-publication history

The pre-publication history for this paper can be accessed here:

http://www.biomedcentral.com/1755-8794/3/20/prepub
